# The population-based prevalence of trachomatous scarring in a trachoma hyperendemic setting: results from 152 impact surveys in Amhara, Ethiopia

**DOI:** 10.1186/s12886-021-01972-w

**Published:** 2021-05-13

**Authors:** Tigist Astale, Caleb D. Ebert, Andrew W. Nute, Mulat Zerihun, Demelash Gessese, Berhanu Melak, Eshetu Sata, Zebene Ayele, Gedefaw Ayenew, E. Kelly Callahan, Mahteme Haile, Taye Zeru, Zerihun Tadesse, Scott D. Nash

**Affiliations:** 1 The Carter Center, Addis Ababa, Ethiopia; 2 F.I. Proctor Foundation, University of California, San Francisco, CA San Francisco, USA; 3 The Carter Center, GA Atlanta, USA; 4 Amhara Public Health Institute, Bahir Dar, Ethiopia

**Keywords:** Trachoma, *Chlamydia trachomatis*, Ethiopia, Survey, Trachomatous scarring

## Abstract

**Background:**

Trachomatous scarring (TS) results from repeated infection with the bacterium *Chlamydia trachomatis.* Pronounced scarring is an underlying cause of trachomatous trichiasis (TT) that can lead to blindness. Since the condition is irreversible, TS in adults has been considered a marker of past exposure to trachoma infection. The aim of this report was to estimate the population-based prevalence of TS within Amhara, Ethiopia, a region with a historically high burden of trachoma.

**Methods:**

District-level multi-stage cluster surveys were conducted in all districts between 2010 and 2015 to monitor the impact of approximately 5 years of trachoma interventions. Approximately 40 households were sampled per cluster and all participants ages ≥ 1 year were graded for the 5 World Health Organization simplified signs. Before each survey round, trachoma graders participated in a 7-day training and reliability exam that included cases of TS. TS prevalence estimates were weighted to account for sampling design and adjusted for age and sex using post-stratification weighting.

**Results:**

Across the 152 districts in Amhara, 208,510 individuals ages 1 year and older were examined for the signs of trachoma. Region-wide, the prevalence of TS was 8.2 %, (95 % Confidence Interval [CI]: 7.7**–**8.6 %), and the prevalence among individuals ages 15 years and older (*n* = 110,137) was 12.6 % (95 % CI: 12.0**–**13.3 %). District-level TS prevalence among individuals ages 15 years and older ranged from 0.9 to 36.9 % and was moderately correlated with district prevalence of TT (*r* = 0.31; *P* < 0.001). The prevalence of TS increased with age, reaching 22.4 % among those ages 56 to 60 years and 24.2 % among those ages 61 to 65 years. Among children ages 1 to 15 years TS prevalence was 2.2 % (95 % CI: 1.8**–**2.8 %), increased with age (*P* < 0.001), and 5 % of individuals with TS also had trachomatous inflammation-intense (TI).

**Conclusions:**

These results suggest that Amhara has had a long history of trachoma exposure and that a large population remains at risk for developing TT. It is promising, however, that children, many born after interventions began, have low levels of TS compared to other known trachoma-hyperendemic areas.

## Background

Trachoma is caused by the bacterium C*hlamydia trachomatis.* Repeated infection and the associated conjunctival inflammation throughout childhood can initiate a scarring process. Conjunctival scarring is a proinflammatory and remodeling response, characterized by disordered subepithelial connective tissue, which can appear as fibrotic bands or sheets [1, 2]. Scarring is thought to be an irreversible process and could be considered a marker for cumulative exposure to *C. trachomatis* infection and associated inflammation [3–7]. Severe enough scarring can cause entropion of the eyelid followed by trachomatous trichiasis (TT), corneal opacity, and eventual blindness.

The SAFE (Surgery, Antibiotics, Facial cleanliness, and Environmental improvement) strategy targets both the early (infective) and late (blinding) stages of trachoma for elimination as a public health problem. The SAFE strategy is warranted for all districts (the administrative unit for health delivery) until the prevalence of trachomatous-inflammation follicular (TF) among children ages 1 to 9 years is < 5 % and the prevalence of TT among individuals ages 15 years and older is < 0.2 % [8]. Prior evidence has demonstrated that conjunctival scarring may still develop and progress over time, even after the prevalence of clinical signs of active trachoma such as TF and trachomatous-inflammation intense (TI) has declined [9–11]. The progression of trachomatous scarring (TS) and subsequent development of TT over time will likely pose a challenge for trachoma elimination and post-elimination surveillance efforts.

Recent population-based surveys have been conducted in the Amhara region of Ethiopia to understand the impact of the SAFE strategy on trachoma [12, 13]. The results of these surveys have demonstrated that the prevalence of both TF and TT remained highly heterogenous throughout the region with many districts still well above the thresholds for elimination of trachoma as a public health problem. Furthermore, it has been demonstrated that considerable *C. trachomatis* transmission was still occurring among children ages 1 to 5 years [12]. While it remains necessary to know the levels of TF and TT for programmatic decisions around mass drug administration (MDA) of antibiotics and surgical services respectively, it may also be important to better understand the underlying pathophysiology experienced by a population continually exposed to endemic levels of trachoma transmission. Understanding the prevalence of TS may help trachoma elimination programs plan interventions to limit disease progression before it reaches the blinding stages and plan future surgical needs in trachoma endemic settings [14].

This report aimed to estimate the prevalence of TS from 152 district-level population-based surveys covering the entire Amhara region. Such data, though rarely reported in many survey methodologies, could provide insights into both the cumulative history of trachoma exposure experienced by this population as well as the future TT burden in formerly endemic districts. A further aim was to detail the epidemiology of TS among children growing up in trachoma endemic communities that have received SAFE interventions.

## Methods

### Study site and study period

SAFE interventions were scaled up between 2007 and 2010 to reach all 152 programmatic districts encompassing the Amhara region [13, 15, 16]. Each district received an average of 5 (range 3 to 7) years of SAFE, including annual MDA with antibiotics, and eyelid surgeries to correct TT. Trachoma impact surveys were conducted once per district between 2010 and 2015 to provide region-wide trachoma prevalence estimates after the SAFE interventions.

### Study design

The methodology for the impact surveys included in this analysis have previously been described [12, 13, 16]. Briefly, a multi-stage cluster random sampling methodology was used to select the study participants for district-level estimates. In the first stage, villages were selected from each district using a population proportional to estimated size sampling method. In the second stage of sampling, segments of households were randomly selected from each village using an existing governmental administrative structure with the goal of 30 to 40 households per segment. All present, consenting individuals within the households in each selected segment were included in these surveys. Individuals aged ≥ 1 year were included in this report.

### Training, data collection, and clinical assessment

Before each survey round, which occurred approximately twice per year, all trachoma graders (integrated eye care workers) participated in a 5 to 7-day training [12, 13, 16]. Graders were required to attend training regardless of the number or date of their previous trainings. Training methods used for these surveys pre-date but were very similar to those used in more current trachoma surveys [17]. Training consisted of classroom sessions to understand the definitions of all 5 World Health Organization (WHO) simplified trachoma signs through slide-deck review, and field-based sessions focused on providing graders experience in the range of presentations trachoma signs can take [18]. Graders were then required to pass a slide-based exam, which included images of all 5 simplified trachoma signs, including approximately 5 images of TS. Graders who passed the slide exam then moved onto a field reliability exam where they were required to grade 50 eyes under field conditions. The “gold standard” for the field reliability exam was the consensus grade of 3 expert graders, typically ophthalmologists, ocular surgeons, or trachoma managers [16, 19]. Those graders who scored an interobserver agreement of ≥ 84 % and kappa ≥ 0.7, compared to the consensus grade of the 3 expert graders for the sign TF joined the survey team [16]. Although the field reliability exam was based on grading TF, graders were also trained on how to identify and grade TS using slide training, slide examination, and field-based practice within TT surgery campaigns that were specifically planned to accommodate the graders’ practice for these surveys.

The primary aim of these surveys was to estimate TF among children ages 1 to 9 years, but all study participants were examined for all 5 WHO simplified signs of trachoma, including TS. Both eyes of all study participants were examined for TS, which was defined as the presence of scarring in the tarsal conjunctiva (scars are easily visible as white lines, bands, or sheets [fibrosis] in the tarsal conjunctiva) [18]. Trachoma grading was performed using binocular loupes with x2.5 magnification under sunlight or using a flashlight. Multiple grading teams worked in each district, and supervisors traveling with grading teams spot-checked grading, particularly during the first 2 weeks of each survey.

Data collection took place on Samsung Galaxy tablets running custom-built software [20]. Data recorders were information technology or biostatistics college graduates who took a 4 to 5-day training and passed a data collection exam using the tablets. All household surveys were conducted in the Amharic language.

### Statistical analysis

All TS prevalence estimates were weighted to account for the probability of sampling at both sampling stages. Furthermore, all TS prevalence estimates reported, district- and region-level, were age-sex adjusted with post-stratification weighting using 5-year age-sex bands from the survey census population [21, 22]. Confidence intervals (CI) were calculated using Taylor linearization and survey procedures (svy package) in Stata, version 15.1 (STATA Corporation, College Station TX, USA), to account for household and village level clustering. Most analyses were focused on individuals ages 15 years and older (standard age group used to estimate TT prevalence), individuals younger than 15 years, and individuals ages 1 to 9 years (standard age group for estimating TF prevalence) [8]. The prevalence of TS concurrent with TF and TI was also estimated. Chi-squared tests were used to evaluate TS prevalence between age group and sex. A Spearman correlation test was performed to examine relationships between TS and TT, TS and TF and TS and TI at the district level. Clustering by household and survey cluster was assessed by calculating the intraclass correlation coefficient (ICC) of TS prevalence with a random-effects model. District-level prevalence was grouped into the categories of < 5 %; 5.0**–**9.9 %; 10**–**14.9 %; > 15 % based on the overall TS prevalence distribution.

## Results

A total of 276,068 individuals were surveyed between 2010 and 2015 in all 152 programmatic districts across the 10 zones of the Amhara region. Among those enumerated, 75.5 % (208,510) of individuals were present and examined for clinical signs of trachoma. The majority of examined individuals, 52.9 % (110,211 respondents), were ages 15 years and older, 35.2 % (73,406 respondents) were children ages 1 to 9 years, and 53.5 % (111,577 respondents) were female.

**Fig. 1:**
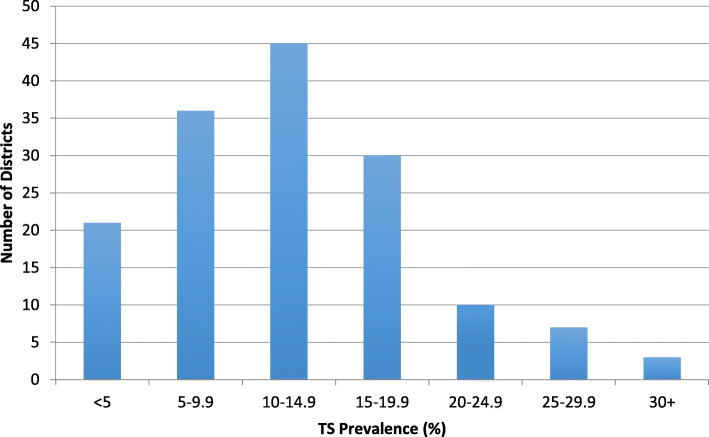
District-level TS prevalence among individuals ages 15 years and older, Amhara, Ethiopia, 2010 to 2015

**Fig. 2:**
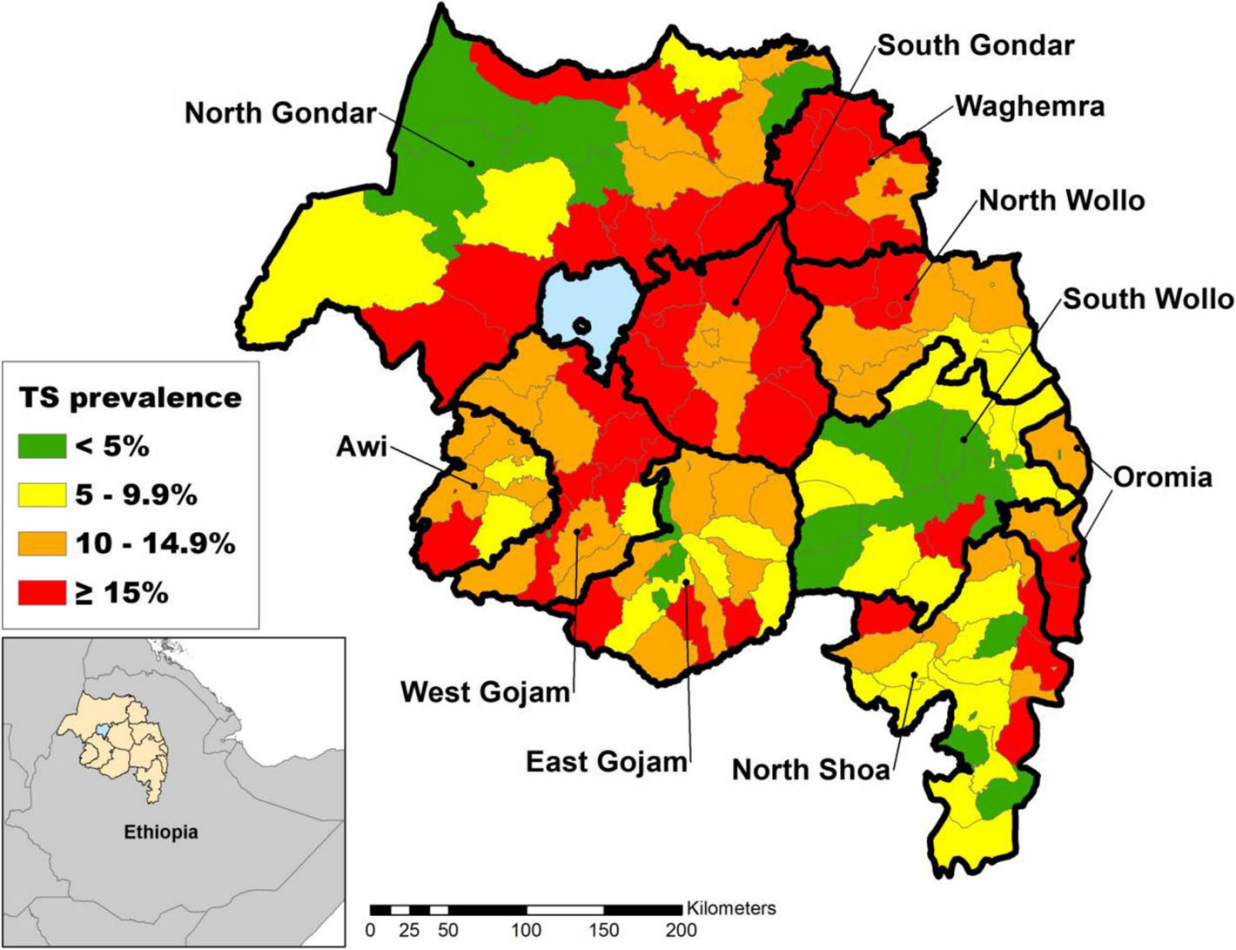
District-level TS prevalence among individuals ages 15 years and older, Amhara, Ethiopia, 2010 to 2015

**Fig. 3:**
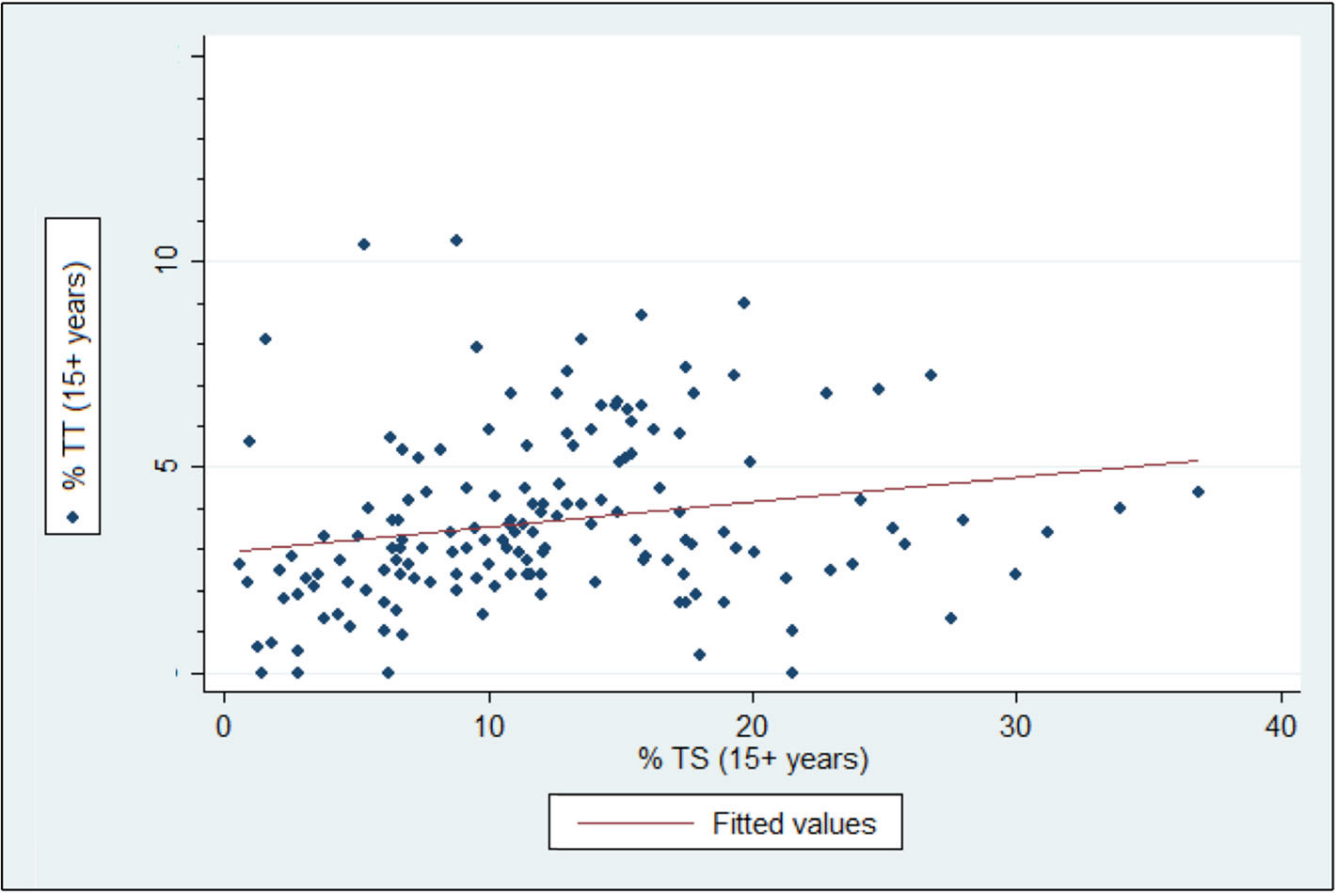
Scatterplot of district-level TS and TT prevalence, Amhara, Ethiopia, 2010 to 2015. N=152 districts, r=0.31

The regional TS prevalence among all ages was 8.2 % (95 % CI: 7.7**–**8.6 %), and the TS prevalence among individuals ages 15 years and older was 12.6 % (95 % CI: 12.0**–**13.3 %). The range of TS prevalence by district (*n* = 152) in this age group was right-skewed and ranged from 0.6 % (95 % CI: 0.1**–**3.4 %) to 36.9 % (95 % CI: 27.2**–**47.8) (Fig. 1). Each of the 10 zones had at least one district with a TS prevalence in the highest category (TS ≥ 15 %), and Waghemra and South Gondar in the central part of the region had the heaviest burden of the high prevalence districts (Fig. 2). The district prevalence of TS was moderately correlated with the prevalence of TT (Spearman correlation [*r*] = 0.31, *P* < 0.001 (Fig. 3)).

**Fig. 4:**
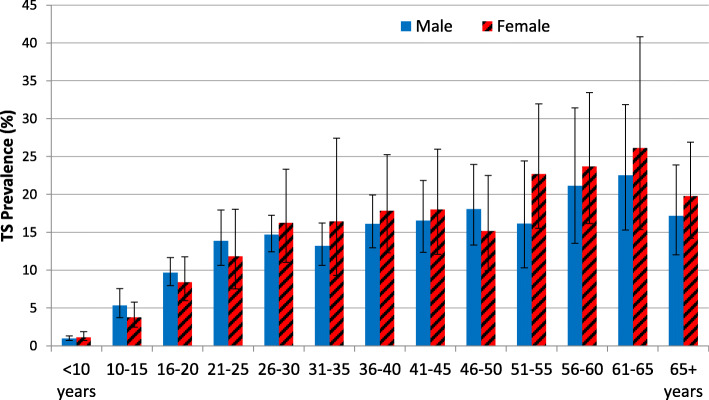
Prevalence of TS by sex and age group, Amhara, Ethiopia, 2010 to 2015

Among individuals, the prevalence of TS increased significantly with age (*P* < 0.0001); the highest prevalence was observed in adults ages 56 to 60 years (22.4 %; 95 % CI: 16.1**–**30.3 %) and ages 61 to 65 years (24.2 %; 95 % CI: 16.4**–**34.2 %) (Fig. 4). Among individuals ages 15 years and older, TS prevalence among females and males was 15.2 % (95 % CI: 11.5**–**19.7 %) and 14.6 (95 % CI: 12.6**–**16.9 %), respectively; the difference was not statistically significant (*P* = 0.73). Among individuals with TT in this age group, 955 (23.3 %) individuals had concurrent TS, and the individual-level correlation between TT and TS was low (*r* = 0.0275; *P* < 0.001).There was some evidence of clustering at the household level with an ICC of 0.24 (95 % CI: 0.23**–**0.26) and little evidence of clustering at the village level with an ICC of 0.08 (95 % CI: 0.07**–**0.10). At the household level, 79.0 % of households did not have any adults with TS, but in 9.3 % of households all of the adults had TS. The remaining 11.7 % of households had at least one but not all adults with TS.

**Fig. 5:**
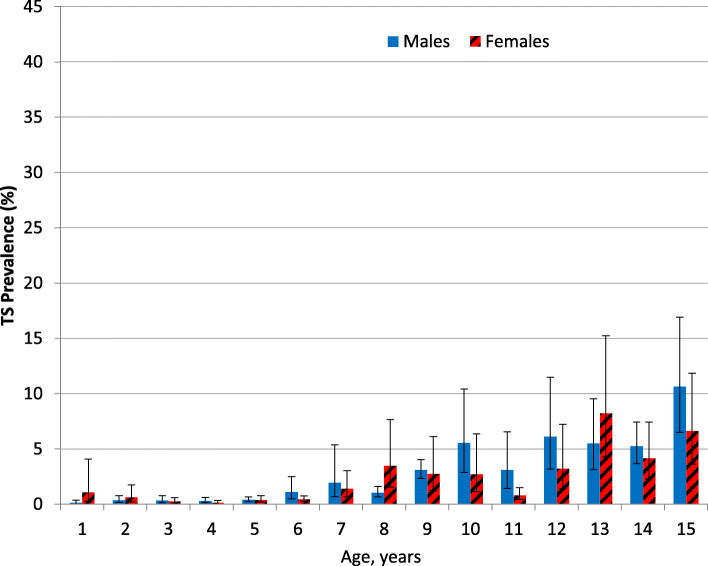
Prevalence of TS by sex and ages 1 to 15 years, Amhara, Ethiopia, 2010 to 2015

**Fig. 6:**
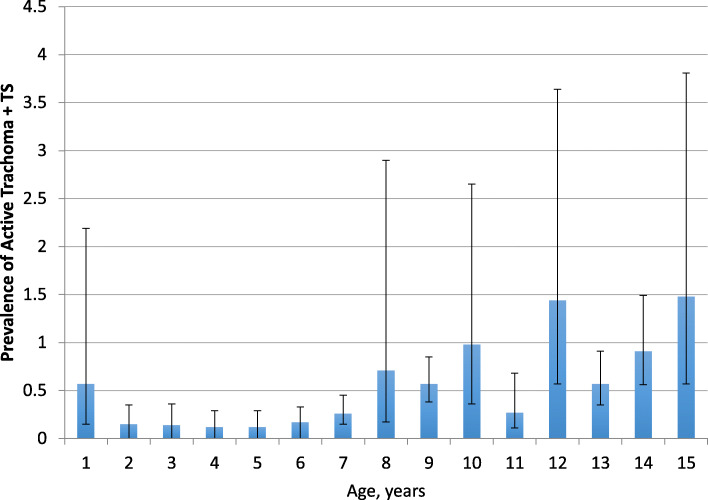
Active trachoma with TS prevalence in ages 1 to 15, Amhara, Ethiopia, 2010 to 2015

The regional TS prevalence among individuals ages 1 to 15 years and individuals ages 1 to 9 years was 2.2 % (95 % CI: 1.8**–**2.8 %) and 1.1 % (95 % CI: 1.0**–**1.2 %) respectively. Moderate correlations were observed between district prevalence TS and district prevalence of TF (*r* = 0.33, *P* < 0.0001) and TI (*r* = 0.52, *P* < 0.0001) among individuals ages 1 to 15 years. Among individuals ages 1 to 15 years, the prevalence of TS increased with age (*P* < 0.001) reaching as high as 10.6 % in 15-year-old males (Fig. 5). Overall, however, the TS prevalence did not differ by sex (*P* = 0.40) in this age group. Among individuals ages 1 to 15 years with TS, 24.3 % also had TF and 4.8 % also had TI. In this age group, the prevalence of concurrent TS and active trachoma (TF and/or TI) was 0.6 % (95 % CI: 0.4**–**0.9 %), while the prevalence of concurrent TS and TF was 0.2 % (95 % CI: 0.04**–**0.9 %) (Fig. 6).

## Discussion

The results of these 152 population-based surveys suggest that the Amhara region has had a long history of intense trachoma exposure, and that a large population remains at risk for developing TT. Future monitoring of incident TT will likely be needed even after the elimination as a public health problem threshold for TT has been reached. It is promising, however, that children ages 1 to 15 years, many born after the MDA program began, have a TS prevalence of 2 %. Currently, programmatic monitoring of scarring is rare, and TS prevalence as a district-level indicator is no longer included in most trachoma survey methodologies. Future longitudinal research is needed to determine whether scarring data can help programs plan future needs for TT services.

Early district-level surveys conducted in Amhara in 2001 and 2003 demonstrated that trachoma was hyperendemic in 5 pilot districts, with a TF prevalence among children ages 1 to 9 years ranging between 49 and 90 % and TT among individuals ages 15 years and older ranging between 2.0 and 5.4 % [15]. In 2006, a zonal-level baseline trachoma survey demonstrated both a high TF (32.7 %) and TT (6.2 %) prevalence region-wide [23]. These results led to the scale-up of the full SAFE strategy in all districts of Amhara. At scale, the Trachoma Control Program was delivering approximately 15 million doses of antibiotic per year as part of its MDA campaigns [13]. Furthermore, as part of TT surgical campaigns throughout the region, over 408,000 surgeries were conducted on TT patients between 2007 and 2015 [13]. Despite this, impact surveys conducted after these interventions demonstrated that many districts still had high levels of TF and *C. trachomatis* infection and that all but 4 districts were still above the elimination as a public health problem threshold for TT [12, 13]. This continued high prevalence of TT observed in many districts may in part be due to incident TT as a result of years of progressive scarring. We found that districts with high levels of TT were more likely to have a concurrently high level of TS. Future surgical services will most likely be needed the most in districts with a high TT burden with concurrently high TS prevalence.

In trachoma-endemic communities, scarring starts to develop in childhood and continues to progress with age. Scarring is thought to be driven by repeated infection with *C. trachomatis*, whereby a higher cumulative burden of infection would lead to increased scarring [24]. It is also believed that scarring may be a result of persistent inflammation [10]. Evidence for this mechanism comes from cohort studies by which progressive scarring was observed in the absence of *C. trachomatis* infection [10, 25]. Within adults with scarring in Amhara, 23 % had observed scarring progression over a 2-year period [10]. Within our surveys, we found that the prevalence of TS increased with age, reaching as high as a quarter of the population among those ages 60 to 64 years. Given the size of Amhara, this represents a large population at risk for scarring progression and incident TT. The Trachoma Control Program in Amhara and in other hyperendemic regions will need to develop surveillance systems to detect and operate incident TT cases. Given the within-household clustering of scarring observed in this report, the Program may expect incident TT to cluster within households as well. All communities within Amhara have received on average 5 years of the SAFE strategy including annual MDA with azithromycin. Azithromycin is an antibiotic shown to be effective against *C. trachomatis* and thought to also have anti-inflammatory properties [26, 27]. It is possible therefore that annual azithromycin distribution will reduce the progression of scarring and possibly the incidence of TT in this population.

In this trachoma endemic setting, TS was detectable in children, although at relatively low levels (≤ 2 %). This low TS prevalence may have been due to the annual antibiotic distribution in communities throughout the region over the last 5 years. Although pre-SAFE TS data from children in Amhara has not been previously published, in neighboring trachoma hyperendemic South Sudan, the prevalence of TS prior to the start of the SAFE strategy was 19.3 % among children ages 1 to 9 years and 31.7 % among children ages 10 to 14 years, measured using the same WHO simplified system [28]. In Guinea Bissau, a country where the TF prevalence pre-SAFE would be considered mesoendemic (22 %), the prevalence of TS among children ages 0 to 5 years and ages 6 to 10 years was 2.7 and 2.8 % respectively [29]. Although comparisons across various settings is difficult due to differences in the assessment of scarring, it appears that the level of scarring observed in Amhara among children is not reflective of a trachoma hyperendemic setting. However, children are clearly still at risk for developing scarring in Amhara since the prevalence of TF, TI, and *C. trachomatis* infection among children ages 1 to 9 years was still 25.9, 5.5, and 5.7 % respectively, and a statistically significant association was observed between age and scarring, even in this limited age range [12, 13]. Prior studies have demonstrated that the presence of TI is associated with the incidence and progression of scarring [4, 10, 24, 30, 31]. We observed that approximately 25 % of children with TS also had TF, and nearly 5 % also had TI, therefore this subset in Amhara may be at particular risk for scarring progression. Although longitudinal studies are needed to know the degree to which scarring is still developing and progressing in Amhara, clearly, further enhanced efforts are needed to reduce the levels of inflammatory trachoma in these communities in order to reducing the burden of future scarring.

These surveys had several limitations. Survey teams graded TS in the field using the WHO simplified grading scheme, and therefore it was not possible to grade scarring severity. As graders were trained to identify bands or sheets that were “easily visible,” it is likely that mild scarring would be missed in this definition. Therefore, our TS estimates may have underestimated the total scarring burden in Amhara, and the correlation between TT and TS may have been attenuated. Evidence for this comes from a study in Amhara showing that 70 % of participants without TT, and 95 % of those with TT had had some degree of scarring using a detailed grading scheme [32]. In research settings, grading using conjunctival photographs has been common and could possibly be considered for programmatic use to allow for more granular grading of scarring. Although the field reliability test was focused on the trachoma sign TF, TS grading was integrated with the survey training, and the trainees had a chance to practice in the field along with TT identification practice that was integrated with TT surgery campaigns. This study was cross-sectional, and therefore could not measure incident or progression of TS, which would be useful indicators for trachoma programs. Currently programmatic monitoring globally no longer includes TS prevalence as an indicator, and longitudinal studies focused on scarring are uncommon [3, 7, 10, 14, 24]. The inclusion of TS estimation within programmatic surveys should be reconsidered as scarring data has recently been used to better understand the history of trachoma within treatment-naïve endemic regions and has further been used to help make MDA treatment decisions under select circumstances [6, 33]. More data on scarring can also provide modelers with empirical data to improve models for the natural history of trachoma and in forecasting incident TT [34, 35].

Overall, a considerable percentage of adults have TS in these trachoma-endemic districts in Amhara, which suggests both a long history of *C. trachomatis* infection and the possibility of continued development of incident cases of TT in the future. Including TS data in the surveillence of trachoma may be useful for the global elimination program. Trachoma programs serving formerly highly endemic areas should continue to develop context-appropriate monitoring systems for incident TT for the foreseeable future.

## Data Availability

All data generated or analyzed during this study are included in this published article.

## Abbreviations

CI: Confidence interval
ICC: Intraclass correlation coefficient
MDA: Mass drug administration
SAFE: Surgery, Antibiotic, Facial cleanliness, and Environmental improvement
R: Spearman correlation
TF: Trachomatous inflammation-follicular
TI: Trachomatous inflammation-intense
TS: Trachomatous scarring
TT: Trachomatous trichiasis
WHO: World Health Organization.
